# Prediction of anticancer peptides against MCF-7 breast cancer cells from the peptidomes of *Achatina fulica* mucus fractions

**DOI:** 10.1016/j.csbj.2015.11.005

**Published:** 2015-12-07

**Authors:** Teerasak E-kobon, Pennapa Thongararm, Sittiruk Roytrakul, Ladda Meesuk, Pramote Chumnanpuen

**Affiliations:** a Department of Genetics, Faculty of Science, Kasetsart University, Bangkok 10900, Thailand; b Department of Zoology, Faculty of Science, Kasetsart University, Bangkok 10900, Thailand; c National Center for Genetic Engineering and Biotechnology, Thailand Science Park, Pathum Thani 12120, Thailand; d Faculty of Dentistry, Thammasat University, Pathum Thani 12120, Thailand

**Keywords:** Peptidomics, Cytotoxic peptides, *Achatina fulica*, Breast cancer, Bioinformatics prediction, Snail mucus

## Abstract

Several reports have shown antimicrobial and anticancer activities of mucous glycoproteins extracted from the giant African snail *Achatina fulica*. Anticancer properties of the snail mucous peptides remain incompletely revealed. The aim of this study was to predict anticancer peptides from *A. fulica* mucus. Two of HPLC-separated mucous fractions (F2 and F5) showed in vitro cytotoxicity against the breast cancer cell line (MCF-7) and normal epithelium cell line (Vero). According to the mass spectrometric analysis, 404 and 424 peptides from the F2 and F5 fractions were identified. Our comprehensive bioinformatics workflow predicted 16 putative cationic and amphipathic anticancer peptides with diverse structures from these two peptidome data. These peptides would be promising molecules for new anti-breast cancer drug development.

## 1 Introduction

Breast cancer is one of the most common diseases in women globally [1]. Several factors make women at high risk of the breast cancer [2]. Early detection and the use of radiation therapy, surgery, and chemotherapeutic drugs including selective estrogen receptor modulators (SERMs) and aromatase inhibitors can reduce invasive breast cancer. However, the patients remain traumatized by the unfavorable side effects [3], [4]. The search for target-specific and less side-effect cancer therapy is still undergoing.

Anticancer peptides have been proved to be effective small molecules (< 50 amino acids) that can act specifically against cancerous cells by either membranolytic mechanism or disruption of mitochondria [5]. The net negative charge of the cancer membrane is an important factor for peptides' selectivity and toxicity [6], as compared to the typically zwitterionic property of non-cancerous eukaryotic membranes. Amphiphilicity levels and hydrophobic arc size allow penetration of these peptides through the cancerous cell membranes and lead to destabilization of the membrane integrity [7], [8]. For example, pleurocidin-like peptides (NRCs) identified from fish could kill breast cancer cells and human mammary epithelial cells by causing membrane damage with subtle harm to human fibroblasts [9]. These cell-penetrating peptides could be used as cancer-specific drug delivery. For example, the non-specific cell-penetrating anticancer peptide buforin IIb was modified to enhance the cancer specificity with no effects on normal cells [10]. This cancer-specific peptide derivative was successfully used to deliver apoptosis-induced antibody into the cancer cells. Distinctively, a peptide SA12 could induce apoptosis on SKBr-3 breast cancer cells by the mitochondrial pathway [11]. Taken together, these physicochemical properties and experimentally validated information were used to develop bioinformatic programs for anticancer peptide prediction and design. AntiCP predicts anticancer peptides by using amino acid composition and binary profiles to develop support vector machine models (SVM) [12]. Another SVM-based program, ACPP, particularly screens for anticancer peptides that contain apoptotic domain [13]. In this regard, these prediction tools will assist high-throughput screening for anticancer peptides from complex peptidomes of an array of natural products.

Giant African snails (*Achatina fulica*) are invasive animals that seriously cause damages to agricultural and ornamental plants worldwide. Only one antimicrobial peptide, namely Mytimacin-AF, was identified from the mucus of *A. fulica*[14]. Mytimacin-AF (9.7 kDa) was a novel cysteine-rich peptide that could inhibit the growth of both fungi and bacteria with little hemolytic effect on human red blood cells. However, we hypothesized that the anticancer peptides from the mucus of *A. fulica* may not be completely revealed. Thus, this study aimed to predict putative anticancer peptides from the most effective HPLC-separated mucous fractions against the breast cancer cell line MCF-7 using mass spectrometric and bioinformatic analysis methods. Our results provide alternative high-throughput screening methods to identify potential anticancer peptides from nearly a thousand peptides within the snail mucus for further validation.

## 2 Experimental procedure

### 2.1 Cell culture

The breast cancer cell line MCF-7 and the kidney epithelial cell line Vero used in this study were kindly provided by the Department of Biochemistry, Faculty of Medicine, Chiangmai University, Thailand and the Genome Institute, National Center for Genetic Engineering and Biotechnology (BIOTEC), Thailand. The cells were cultured and passaged in Dulbecco's Modification of Eagle's Medium (DMEM, Gibco-RBL, Life Technologies, NY) supplemented with 10% Fetal Bovine Serum (FBS, Hyclone, Thermo Fisher Scientific Inc., USA), 1% Penicillin–Streptomycin (PAA, Laboratories GmbH, Austria) and 1% Amphotericin B (PAA, Laboratories GmbH, Austria). The cells were maintained at 37 °C in 95% relative humidified atmosphere containing 5% CO_2_. Cell growth was measured under a light microscope and 80% confluence of the cells was used in all experiments.

### 2.2 Separation of *A. fulica* mucus by HPLC

The snail mucus samples were collected from adult *A. fulica* by intermittent irritation in an ultrasonicating bath at 30 °C sporadically. The crude mucous samples were separated by ZORBAX 300SB-4.6 × 150 mm C18 column, 5 μm, (Agilent, Palo Alto, CA) with Agilent® 1200 system using methanol–water (50:50) with 0.1% trifluoroacetic acid (adjusted from [15]) as mobile phase and the flow rate was 0.30 ml/min. Numbers of the HPLC peaks were used to determined numbers of the fractions. Six HPLC-separated mucous fractions were collected manually and named as F1, F2, F3, F4, F5 and F6 fractions. All HPLC fractions and the crude mucus were concentrated by freeze-drying at − 100 °C and kept at − 20 °C until use.

### 2.3 Determination of cytotoxicity of the mucous fractions by MTT assay

Cell viability count was performed using 3-(4,5-dimethylthiazol-2-yl)-2,5-diphenyltetrazolium bromide (MTT) assay [16]. Cells were seeded at 2 × 10^4^ cells per well (200 μl/well) in 96-well tissue culture plates and allowed cells to adhere for 24 h at 37 °C in the CO_2_ incubator. The culture medium was then replaced with 200 μl/well of the fresh medium for the control group and 200 μl/well of the fresh medium containing the same concentration (1000 μg/ml) of the crude mucus or the six HPLC-separated fractions. After 72 h incubation, 50 μl/well of tetrazolium bromide salt solution (2 mg/ml of stock in phosphate buffered saline, PBS) was added into 150 μl of the cell suspension. Four hours before completion, the reaction mixture was carefully taken out and 200 μl/well of dimethyl sulfoxide or DMSO (Sigma, USA) was added to each well before the addition of 25 μl/well of Sorensen's glycine buffer (Research Organics, USA). The optical densities (OD) were measured at 570 nm using microplate reader (Tecan Sunrise, Switzerland). Finally, the highest effective anti-breast cancer fraction with the lowest percentage of cell viability was then selected for further analysis.

Cytotoxicity of the mucous fractions against the MCF-7 and Vero cells was compared by slightly modified the above-described method due to the limited quantity of the fractions. The cells were seeded at 4 × 10^3^ cells per well in 96-well tissue culture plates and allowed cells to adhere for 24 h at 37 °C in the CO_2_ incubator. The culture medium was then replaced with 100 μl/well of the fresh medium for the control group and 100 μl/well of the fresh medium containing three concentrations (1, 10 and 100 μg/ml) of the crude mucus, the F2, and F5 fractions. After 24 h incubation, 25 μl/well of tetrazolium bromide salt solution (5 mg/ml of stock in PBS) was added to the cell suspension. Four hours before completion, the reaction mixture was carefully taken out, and 100 μl/well of DMSO was added to each well. The optical densities were measured at 570 nm.

### 2.4 Statistical analysis of the MTT assay

The results were presented as mean ± sem (standard error of mean) or mean ± sd (standard deviation). The parameters were analyzed with one-way analysis of variance (One-way ANOVA) followed by Sidak's multiple comparisons test. Statistical analysis was conducted with Graphpad Prism version 6.0 for Windows (Graphpad software, San Diego, California, USA). Significant levels were considered at p < 0.05 and highly significant level at p < 0.01 comparing with control group.

### 2.5 Mass spectrometric analysis of the selected cytotoxic fractions

#### 2.5.1 Sample fractionation

Individual selected fractions were fractionated based on their molecular size using Macrosep® 3 K, 10 K and 50 K Omega centrifugal devices (Pall Life Sciences, USA) into four sub-fractions: lower than 3 kDa, between 3 and 10 kDa, between 10 and 50 kDa, and larger than 50 kDa sub-fractions. These sub-fractions were mixed well with two volumes of cold acetone and incubated overnight at − 20 °C. The mixture was centrifuged at 10,000 ×*g* for 15 min, and the supernatant was discarded. The pellet was freeze-dried and stored at − 80 °C before use.

#### 2.5.2 Determination of protein/peptide concentration by lowry method

The pellets were resuspended in 0.15% Sodium Deoxycholic acid (DOC) or 0.5% SDS and determined protein concentration by Lowry method [17]. The absorbance at 750 nm (OD_750_) was measured, and the protein concentration was calculated using the standard curve, plotted between OD_750_ on Y-axis and BSA concentration (μg/ml) on X-axis.

#### 2.5.3 In-solution digestion

Each protein sub-fractions were hydrolyzed by trypsin at an enzyme to the protein ratio of 1:50 at 37 °C for 24 h, except the lower than 3 kDa sub-fraction. The peptides were dried by vacuum centrifuge and kept at − 80 °C for further mass spectrometric analysis.

#### 2.5.4 HCTultra LC–MS analysis of the peptidomes

Peptide solutions were analyzed using an HCTultra PTM Discovery System (Bruker Daltonics Ltd., U.K.) coupled to an UltiMate 3000 LC System (Dionex Ltd., U.K.). Peptides were separated on a nanocolumn (PepSwift monolithic column 100 μm i.d. × 50 mm). Eluent A was 0.1% formic acid, and eluent B was 80% acetonitrile in water containing 0.1% formic acid. Peptide separation was achieved with a linear gradient from 10% to 70% B for 13 min at a flow rate of 300 nl/min, including a regeneration step at 90% B and an equilibration step at 10% B, one run took 20 min. Peptide fragment mass spectra were acquired in data-dependent AutoMS (2) mode with a scan range of 300–1500 *m*/*z*, 3 averages, and up to 5 precursor ions selected from the MS scan 50–3000 *m*/*z*.

#### 2.5.5 Identification of peptide sequences

The MS/MS data from LC–MS were submitted to database search using the Mascot software (Matrix Science, London, U.K., [18]). The peptide sequence data was searched against the NCBI database for protein identification. Database interrogation was; taxonomy (other metazoans); enzyme (trypsin); variable modifications (carbamidomethyl, oxidation of methionine residues); mass values (monoisotopic); protein mass (unrestricted); peptide mass tolerance (1 Da); fragment mass tolerance (± 0.4 Da), peptide charge state (1 +, 2 + and 3 +), max missed cleavages (1) and instrument = ESI-TRAP. Proteins considered as identified proteins had at least one peptides with an individual Mascot score corresponding to p < 0.05. The resultant peptides with the rank of ion matches (pep_rank) equivalent to one were considered significance.

### 2.6 Bioinformatic prediction of anticancer peptides

**Fig. 1:**
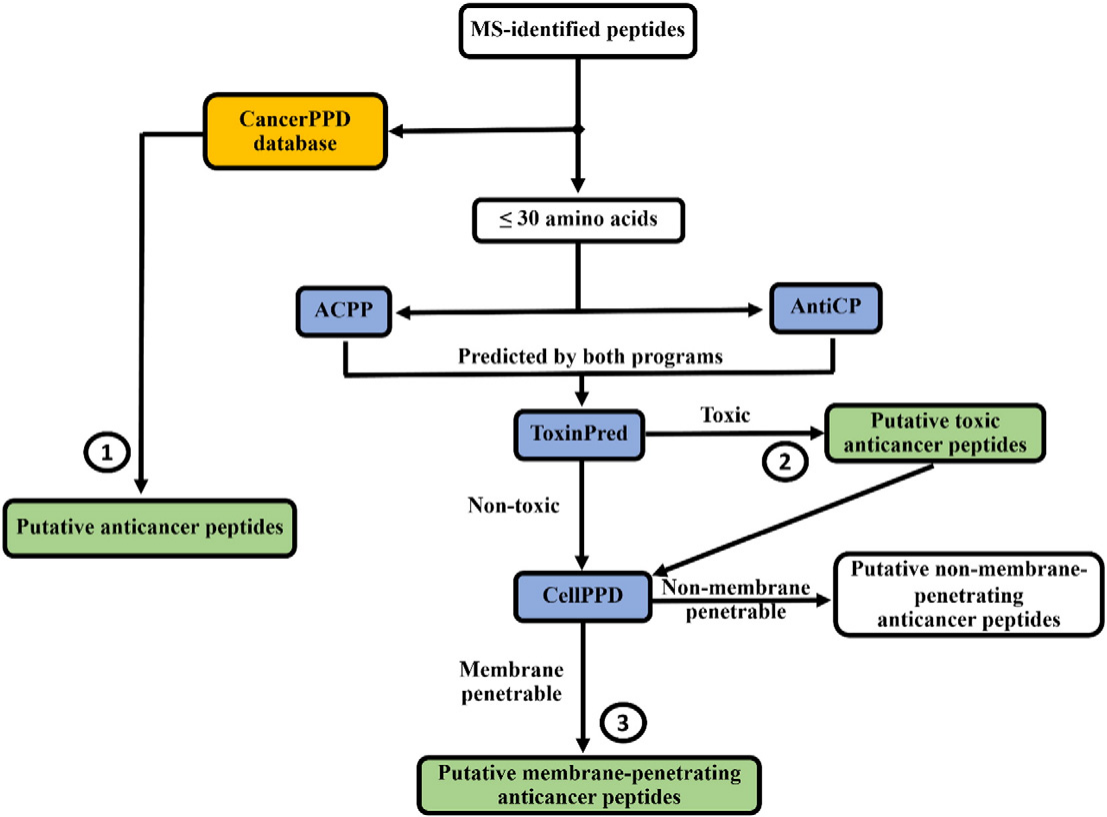
Workflow for bioinformatic prediction of putative anticancer, cytotoxic and membrane-penetrating peptides (labeled with numbers 1, 2 and 3) obtained from peptidomic analysis of *A. fulica* mucous fractions. Details were described in the text. CancerPPD (yellow box) was a database of anticancer peptides. ACPP, AntiCP, ToxinPred and CellPPD were the bioinformatics prediction programs (blue boxes).

The identified peptides from the fractions F2 and F5 were then blasted against a database of anticancer peptides (cancerPPD, [19]). These peptides were screened for putative anticancer peptides by using consensus prediction. Each peptide was submitted for the prediction of two programs; ACPP [13] and AntiCP [12] for anticancer peptide prediction based on amino acid composition, conserved features and physicochemical properties. The hydrophobicity, hydrophilicity, amphiphaticity and hydrophathicity scores were computed by AntiCP and their means were statistically compared between the anticancer and non-anticancer peptides by using an R package for independent two sample t-test. Peptides positively predicted by both programs were considered putative anticancer peptides. These peptides were checked for toxicity by submission to ToxinPred for the prediction of toxic peptides [20]. The predicted anticancer peptides were subjected to CellPPD for prediction of cell penetrating properties [21]. These peptides were classified into putative membrane-penetrating and non-membrane-penetrating anticancer peptides (Fig. 1). If the number of predicted anticancer peptides was very high, the cutoff score (from 0.5–1.0) was then applied to narrow the list down to a short list of those with the high prediction scores. Structures of these putative anticancer peptides were then modeled by the PEP-FOLD program version 1.5 [22]. Structural similarity was used to classify these predicted peptide structures.

## 3 Results and discussion

**Fig. 2:**
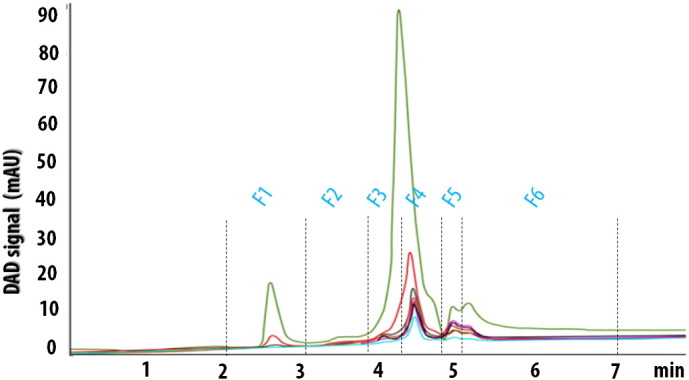
HPLC chromatogram shows retention times (minutes) of the six separated fractions of the *A. fulica* mucus. The signal according to the diode array detector (DAD) is represented in milli-arbitary unit (mAU). The fractions were scanned at different ultraviolet wavelengths; 210 (pink), 220 (green), 230 (purple), 250 (red), 280 (brown green) and 290 (blue) nm.

The *A. fulica* mucus was successfully collected by intermittent irritation and separated into six fractions by the C18-reverse phase HPLC system using methanol–water mobile phase. All six fractions were manually collected and named as F1, F2, F3, F4, F5 and F6 fractions as shown in Fig. 2. The fraction was scanned between 210 to 290 nm which was a suitable range for peptide bond and aromatic amino acid absorptions. The sample showed the highest signal when scanning at 220 nm, but revealed two distinguished peaks at the absorbance of 210, 230, 280 and 290 nm. Thus, these two peaks were collected in the F3 and F4 fractions. The lowest signal peak was collected in the F2 fraction. Other three moderate signal peaks were collected in the F1, F5 and F6 fractions.

### 3.1 Selection of highly cytotoxic mucous fractions against MCF-7 cells

**Fig. 3:**
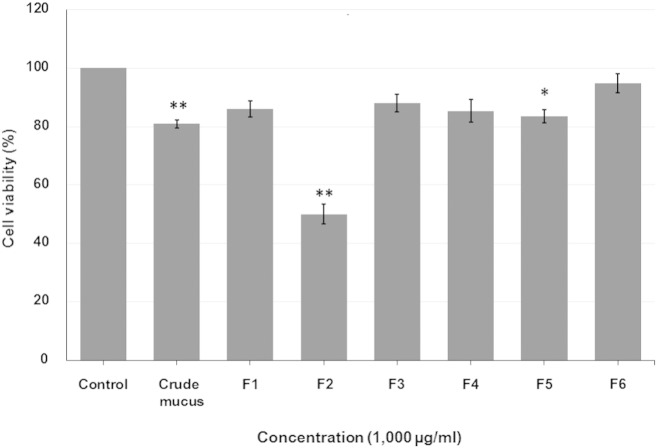
Percentages of the MCF-7 cell viability after 72 h treatment with *A. fulica* crude mucus and six HPLC-separated fractions (F1, F2, F3, F4, F5 and F6) at the concentration of 1000 μg/ml. The cell viability was measured by the MTT assay. Control was the untreated cells. Error bars represented the SEM, * and ** represented levels of significance at p < 0.05 and p < 0.01.

Cytotoxicity of the *A. fulica* crude mucus and six HPLC-separated fractions (F1, F2, F3, F4, F5 and F6) on the MCF-7 cells were evaluated by the MTT assay to measure cytotoxic effect on cell viability. Results showed that fractions F1, F3, F4 and F6 did not significantly induce cell death (p > 0.05) comparing to the control group. The treatments of these four fractions resulted in more than 85% of the viable cells. Oppositely, the crude mucus, fractions F2 and F5 significantly had cytotoxic effects to the MCF-7 cells (p < 0.05) comparing to the control group. The F2 fraction showed the highest cytotoxic effect (50.03 ± 3.38% of viable cells) followed by crude mucus and the F5 fraction (80.91 ± 1.38 and 83.61 ± 2.26%, respectively) (Fig. 3).

**Fig. 4:**
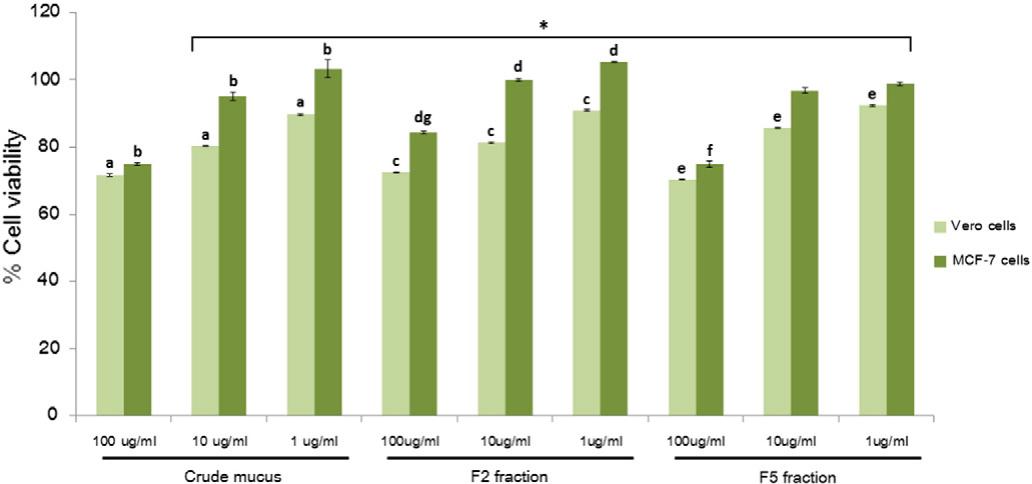
Percentages of the Vero and MCF-7 cell viabilities after treatment with *A. fulica* crude mucus and the F2 and F5 fractions at the concentration of 1, 10 and 100 μg/ml. The cell viability was measured by the MTT assay. Control was the untreated cells. Error bars represented the SD, * represented statistical difference between the viability of Vero and MCF-7 cells at the significance of p < 0.05. a, b, c, d, e and f: significant difference between concentrations of the crude mucus (a and b), the F2 (c and d) and F5 (e and f) fractions, respectively, p < 0.05. g represented the significant difference (p < 0.05) of the F2 fraction from the crude mucus and the F5 fraction at 100 μg/ml.

Cytotoxicity against the MCF-7 and Vero cells was further compared by varying the concentration of the crude mucus, the F2 and F5 fractions to 1, 10 and 100 μg/ml. Results in Fig. 4 showed that the increased concentration of crude mucus and the F2 fraction significantly reduced the viability of both cell types to 72–84% (p < 0.05). The F5 fraction at 100 μg/ml significantly decreased the viability for both Vero and MCF7 cells but showed no different reduction at 1 and 10 μg/ml. The crude mucus and these two fractions significantly suppressed cell viability of the Vero cells more than those of the MCF-7 cells (p < 0.05), except the crude mucus at 100 μg/ml. The Vero cells were insignificantly affected by these three fractions at 100 μg/ml (p < 0.05), whereas the viability of the MCF-7 cells were significantly interfered by the crude mucus and the F5 fraction more than the F2. Although this experiment was not conducted at the concentration of 1000 μg/ml as described in the previous section (Fig. 3) due to limited amount of the HPLC-separated fractions. The percentages of cell viability from these two independent experiments was unable to compare. However, the authors observed similar pattern of the cell viability reduction between the crude mucus and the F5 fraction. For the F2 fraction, the cytotoxic activity could substantially depend on its concentration. These results leaded to the hypothesis that peptides or proteins within the F2 and F5 fractions could have different cytotoxic mechanisms against the cells. Modification of these peptides or proteins could enhance specificity to the MCF-7 cells. Thus, the F2 and F5 fractions were selected for peptidomic analysis.

### 3.2 Peptidomic identification of the selected mucous fractions

The fractions F2 and F5 showed in vitro inhibitive effects on the MCF-7 and Vero cells. These two fractions were then further separated by molecular masses into four sub-fractions: lesser than 3 kDa, between 3 kDa and 10 kDa, between 10 kDa and 50 kDa and larger than 50 kDa. While the < 3 kDa sub-fraction was directly subjected to the LC–MS/MS analysis, the other three sub-fractions were tryptic digested in solution prior mass spectrometric analysis. The MASCOT search of the results against the NCBI database did not give significant matched proteins, despite 424 and 404 peptides were detected. Limited genomic and proteomic information of gastropods in the NCBI database caused this unsuccessful protein identification. If the database did not contain the unknown peptides, it should at least find the closest homologue. This finding was similar to an annotation of a non-model gastropod *Nerita melanotragus* transcriptome that only 15–18% of the contigs were assigned with a putative function [23]. However, amino acid sequences of these peptides were further analyzed by peptidomics. The majority of these peptides (82–84%) were less than 10 kDa. These peptides were blasted against the anticancer peptide database CancerPPD [19] to find similarities to any known anticancer peptides, but no significant hits were found.

Four bioinformatic programs; ACPP, AntiCP, CellPPD and ToxinPred, were then used to predict putative anticancer peptides from the mass spectrometric-detected peptides using our designed workflow in Fig. 1. Forty-three putative anticancer peptides were firstly predicted from the F2 and F5 fractions by both ACPP and AntiCP (Table 1) with the positive prediction scores.

**Fig. 5:**
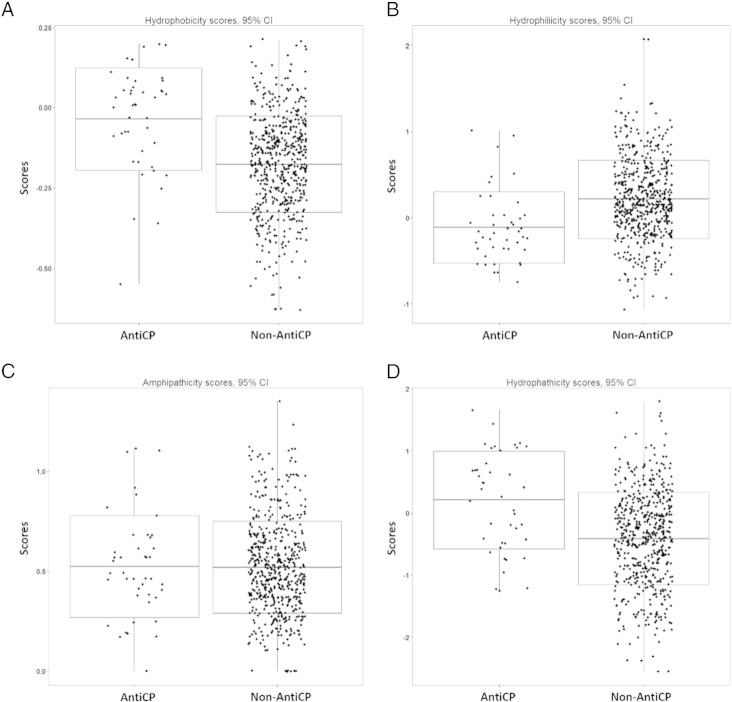
Physicochemical properties of the putative anticancer peptides compared with the non-anticancer peptides from fractions F2 and F5 of *A. fulica* mucus. Hydrophobicity (A), hydrophilicity (B), amphiphaticity (C) and hydrophathicity (D) scores of these peptides were computed by AntiCP. The boxplots were drawn by R program. The scores were shown in the black dots. The uppermost and lowermost ends represented maximum and minimum scores. The middle line indicated means and the two paralleled lines showed standard deviations.

Of these 43 peptides, 23 (5.4%) peptides were from the F2 fraction and 20 (4.9%) peptides were from the F5 fractions. The majority of the putative anticancer peptides of the F2 and F5 fractions were less than 10 kDa with the range of sizes between 5 to 25 amino acids. Analysis of physicochemical properties of the 43 putative anticancer peptides by AntiCP showed a higher average score of hydrophobicity (p < 0.01) and a lower average score of hydrophilicity (p < 0.01) in compared to the average scores of 785 non-anticancer peptides from both HPLC-fractions (Fig. 5A and B). These putative cationic anticancer peptides revealed a greater score of hydropathicity (p < 0.01) meaning that they were more hydrophobic (Fig. 5D). Positive scores of amphipathicity were averagely similar to the non-anticancer peptides (Fig. 5C). This amphiphatic property indicated the ability of these peptides to bind and penetrate the breast cancer cell membrane [7], [8]. However, this property was not clearly separated between the two groups in this study.

Five of these peptides from both F2 and F5 fractions had cell penetrating ability as predicted by CellPPD and only one peptide from the F2 fraction was toxigenic as predicted by ToxinPred. The CellPPD and ToxinPred programs helped predicting specific characteristics of these peptides. The results could partially explain the cytotoxic mechanisms of the F2 and F5 fractions against the Vero and MCF-7 cells. More complicated situations could be that different peptides worked in combination or the cytotoxicity was also concentration-dependent. The predictions could select candidate peptides for further experimental validations.

**Fig. 6:**
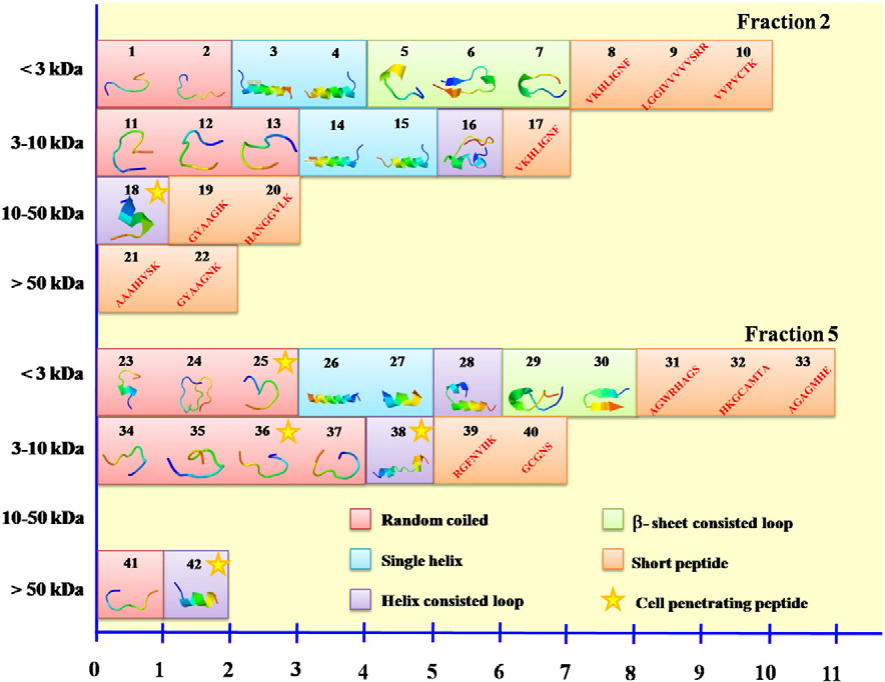
Structural predictions of the putative anticancer peptides obtained from the F2 and F5 fractions of the *A. fulica* mucus. These two fractions were separated into four sub-fractions: < 3 kDa, 3–10 kDa, 10–50 kDa and > 50 kDa. The peptide contents of these sub-fractions were analyzed by protein assay and mass spectrometry. All sub-fractions were trypsinized except the < 3 kDa sub-fraction. Putative anticancer peptides and their structures were predicted by bioinformatic programs and shown in this figure. The structures of these peptides were classified into five categories: random coiled, a single helix, helix-consisted loop, β-sheet-consisted loop and short peptide. Putative peptides predicted from the F2 fraction were displayed on the top half, while those of the F5 fraction were shown on the bottom half. The asterisks indicated the cell-penetrating peptides.

Structures of these 43 peptides were simple and could be classified into five structural categories: random coiled, a single helix, helix-consisted loop, β-sheet-consisted loop and short peptide (Fig. 6). The short putative anticancer peptides (less than 9 amino acid residues) were predominantly observed in the F2 fraction, whereas the F5 fraction contained more of the random coiled peptides. Similar observation [24] was shown in the classification of antimicrobial peptides from the ADP database that the majority of the antimicrobial peptides had no known structure (40%). Notably, most of the known structures of these peptides were β-sheet structure with disulfide bonds and helical structure.

**Fig. 7:**
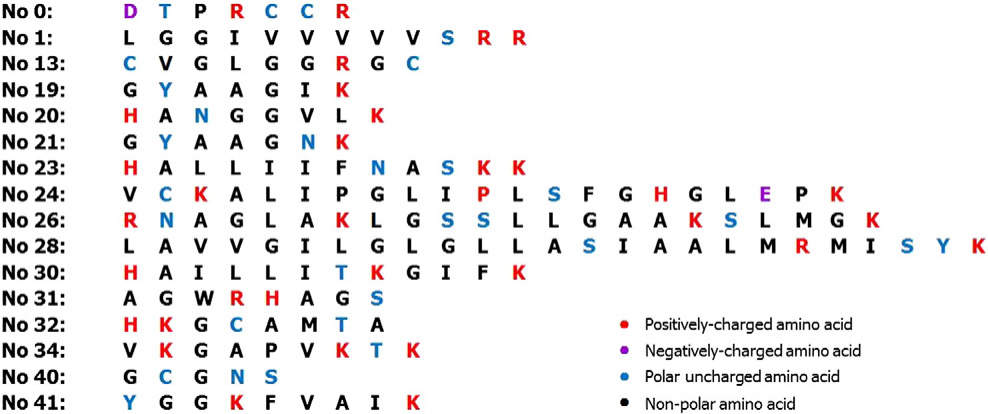
Amino acid constituents of 16 significantly predicted putative anticancer peptides from the F2 and F5 fractions of the *A. fulica* mucus. Colors represented amino acid properties as positively-charged, negatively-charged, polar and non-polar.

As the predictions relied on the support-vector-machine (SVM) score for confident determination of the anticancer peptides by ACPP and AntiCP programs, the threshold of 0.9 was optimally set for the putative anticancer peptide candidates. Applying this stringent criterion narrowed the result down to 16 significantly putative cationic anticancer peptides (Table 1 and Fig. 7). Four short-length (No 0, DTPRCCR; No 19, GYAAGIK; No 20, HANGGVLK and No 21, GYAAGNK) and two random-coiled (No 1, GGPIAAPEASK and No 13, CVGLGGRGC) peptides were from the F2 fraction, while ten peptides were from the F5 fraction which contained one single helix (No 26, RNAGLAKLGSSLLGAAKSLMGK), one helix-consisted loop (No 28, LAVVGILGLGLLASIAALMRMISYK), one β-sheet-consisted loop (No 30, HAILLITKGIFK), four random-coiled (No 23, HALLIIFNASKK; No 24, VCKALIPGLIPLSFGHGLEPK; No 34, VKGAPVKTK and No 41, YGGKFVAIK) and three short peptides (No 31, AGWRHAGS; No 32, HKGCAMTA and No 40, GCGNS) (Fig. 6). Of these 16 peptides, one random-coiled peptide (No 23) had scores beyond 0.9 from both programs. A recent review had shown that the alpha-helical cationic anticancer peptides were promising anticancer agents with unique mechanisms of cytoplasmic membrane disruption leading to necrosis and apoptotic induction by interruption of mitochondrial membrane [25]. Alteration of amino acid residues of these predicted peptides to improve their net charge, hydrophobicity and helicity could enhance their specific binding to the cancer cells [8]. Therefore our findings could be further optimized by a de novo peptide design and modification.

In discussion, this study examined cytotoxicity of the crude *A. fulica* mucus and six HPLC-separated fractions against the breast cancer cell line MCF-7 by using the MTT assay and found that the F2 and F5 fractions significantly induced cell death. These fractions also affected the normal Vero cells. As the MTT assay measured cell viability by colorimetric assessing the activity of NADH-dependent oxidoreductase, this study showed the anticancer activity of the *A. fulica* mucus and the cytotoxic compounds were potentially located in the F2 and F5 fractions. By scanning each HPLC-separated fractions at 210–290 nm, the absorbance at 220 and 280 nm of proteins was clearly observed. This was supported by the protein assay and the mass spectrometric analyses of the F2 and F5 fractions. Therefore, we ascertained that these fractions contained peptides. Previous study suggested that the snail mucus contained complex mixture of proteoglycans, glycosaminoglycans, glycoproteins, hyarulonic acid, small peptides and metal ions [26]. As the crude mucus samples were not processed through enzymatic digestion or heat treatment, the proteoglycan, glycosaminoglycan and hyarulonic acid contents of the mucus could almost be precipitated after centrifugation, yielding only small soluble peptides, glycoproteins and other small compounds.

The cytotoxic effect of the peptide/protein contents within the F2 and F5 fractions was initially hypothesized as the result of a previously characterized glycoprotein enzyme of *A. fulica* mucus, named achacin. Kanzawa et al. found that the purified achacin from *A. fulica* mucus could induce death of HeLa cells at the IC_50_ of 10 μg/ml [27]. Achacin (59 kDa) is an l-amino acid oxidase which catalyzes oxidative deamination of l-amino acids to α-keto acids, hydrogen per oxide (H_2_O_2_) and ammonia (NH_3_) [28]. The production of H_2_O_2_ could induce apoptosis by causing membrane blebbling. Achacin could also reduce amount of free amino acids and triggered the caspase pathway causing chromatin condensation and DNA fragmentation [27], [29]. Although an amino acid sequence of achacin is available in the protein database, proteomic analyses of these two fractions did not detect any peptide fragments of achacin. Therefore, other small peptides could be the cause of these cytotoxic effects.

The peptidomic contents of the F2 and F5 fractions were sub-fractioned by molecular sizes before elucidated by the mass spectrometric analysis which revealed considerable numbers of small peptides. Little information about these various small peptides have been known because the majority have focused on the large constituents of the *A. fulica* mucus. This study predicted approximately 5% of these peptides were putative anticancer peptides and most of them were smaller than 10 kDa, considerably tinier than achacin. Further classification of these anticancer peptides after prediction of peptide folding showed five structural groups. Bringing together these two parts so that 16 small cationic amphipathic peptides with variable structures were finally predicted from the F2 and F5 fractions. These peptides were shorter than a previously reported cysteine-rich antimicrobial peptide (80 amino acids, 9.7 kDa) from the mucus of *A. fulica*, named mytimacin-AF [14]. Mytimacin-AF showed antimicrobial activity with the MIC of 1.9 μg/ml and little hemolytic activity against human red blood cells. Amino acid sequences of our predicted anticancer peptides did not significantly share similarity with mytimacin-AF. Therefore, this study has identified the smallest anticancer peptides from the mucus of *A. fulica* for the first time. These anticancer peptides could play innate immune protective roles in the *A. fulica*. Biochemical synthesis and modification of these peptides are in progress to increase the cytotoxic effect against the breast cancer cells. Moreover, a single peptide may not solely inhibit the growth of the MCF-7 cells, combinatorial effects of anticancer peptides and proteins will be further examined.

Several molluscan small bioactive peptides have been previously reported since 1996 in *Mytilus* sp., *Crassostrea* sp., *Ruditapes philippinarum*, *Biomphalaria glabrata*, *Argopecten irradians*, *Bathymodiolus azoricus*, *Chlamis farreri*, *Haliotis* sp., *Mercenaria mercenaria*, *Littorina littorea*, *Hyriopsis cumingii*, *Venerupis philippinarum* and *A. fulica*[30], but only a few peptides were identified from gastropod species such as antimicrobial peptides in *B. glabrata*, littorein in *L. littorea* and Mytimacin-AF in *A. fulica*[14]. Most of these peptides were small cationic cysteine-rich amphipathic molecules. The 16 predicted anticancer peptides in this study were also small cationic molecules with amphipathic properties. A proline-rich antimicrobial peptide from the Chilean scallop *A. purpuratus* was an example of non-cysteine-rich bioactive peptides [30]. The anticancer peptides are potential targets over other chemicals for new therapeutic agents due to their low molecular masses, simple structures, specific cytotoxicity to cancer cells over normal cells, various routes of administration, and lower the risk of drug resistance induction [31]. These peptides have diverse mechanisms of action as reviewed by Mulder et al. [31]. The cationic anticancer peptides can bind to the negatively-charged membrane of the cancer cells and disrupt the membrane stability and fluidity, such as pore formation by the carpet model or barrel-stave model, or increase of calcium ion influx, leading to cell death. Some of these peptides may inhibit cancer cell growth by modification of lysosomal membrane, enhancement of proteasome activity, induction of mitochondrial pathway of apoptosis by the caspase cascade, activation of immune modulatory pathway, and inhibition of DNA replication-relating genes interfering the cell cycle. Detailed analyses as well as residue modification will be conducted for better understanding the cytotoxic mechanisms of these 16 peptides. However, tiny amount of the F2 and F5 fractions as well as their sub-fractions have been obtained, exhaustive experimental screening of the active peptides within these fractions are hardly difficult. Synthetic peptides would be an alternative option for further analysis.

In summary, anticancer property of small peptide contents within the F2 and F5 HPLC-separated fractions from *A. fulica* mucus against the breast cancer cell line MCF-7 was shown. Mass spectrometric and bioinformatics analyses characterized and predicted relatively small cationic amphipathic putative anticancer peptide candidates in these fractions, for the first time. These peptides will be promising targets for new anticancer drug development.

## Author contributions

All authors read and approved the final manuscript. T. E-kobon analyzed data and performed bioinformatics predictions and wrote the manuscript; P. Thongaram collected mucus fraction and conducted cytotoxicity test; P. Chumnanpuen designed experiments, performed HPLC analysis, peptidomic analysis and revised the manuscript; L. Meesuk assisted in cell culture and cytotoxicity test; S. Roytrakul performed LC–MS/MS analysis and assisted in cytotoxicity test.

## Conflicts of interest

The authors declare no conflict of interest.

## Figures and Tables

**Table 1:**
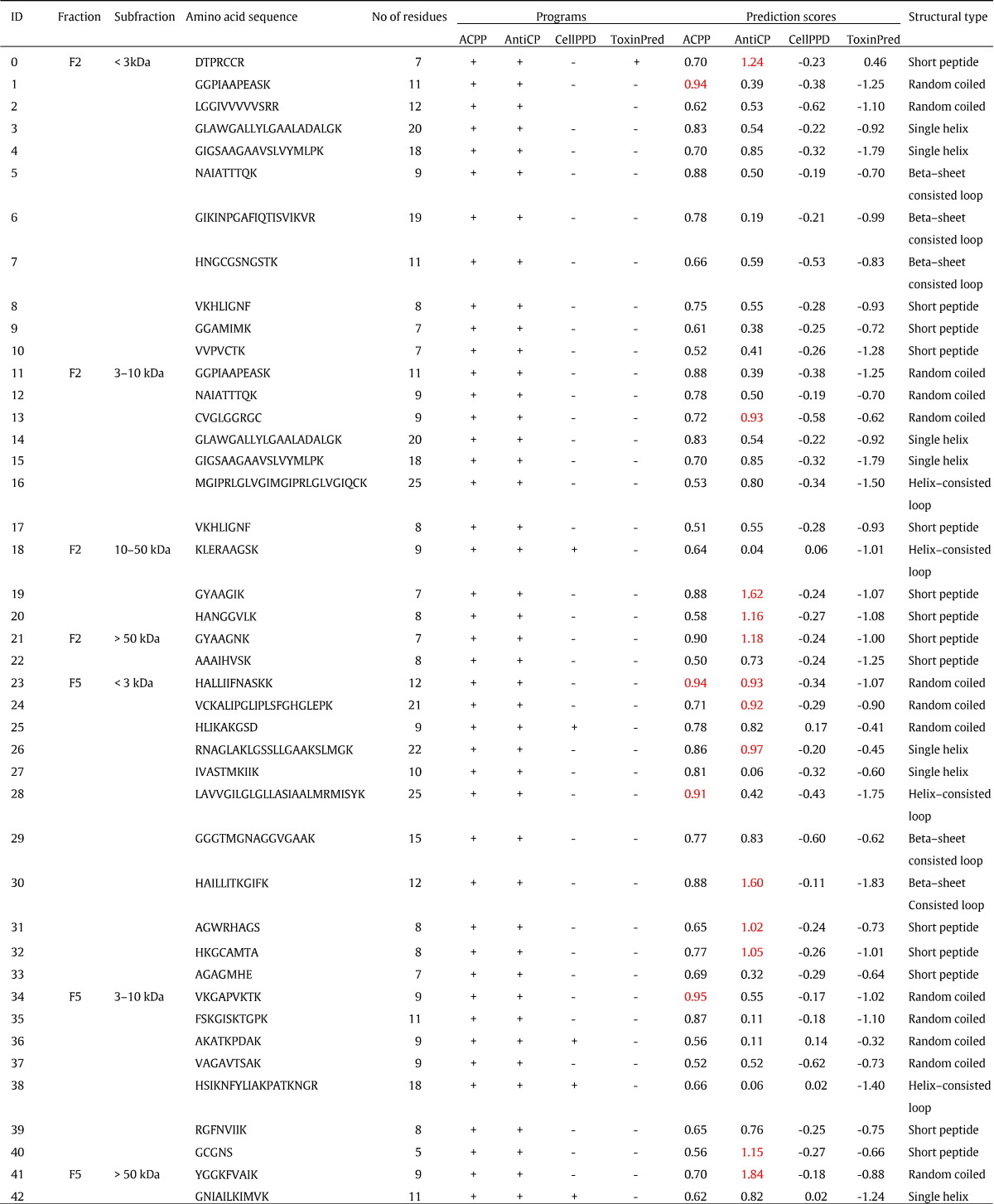
Details of putative anticancer peptides predicted from the F2 and F5 fractions of the *A. fulica* mucus by using four bioinformatics programs. Each fraction consisted of four sub-fractions: < 3 kDa, 3–10 kDa, 10–50 kDa and > 50 kDa. Predictive scores and amino acid sequence for each peptide were also shown. These peptides were structurally classified into five groups including random coiled, a single helix, helix-consisted loop, β-sheet-consisted loop and short peptide. The SVM scores above the threshold of 0.90 are highlighted in red. The 16 significantly putative anticancer peptides are showed in bold amino acid sequences.
